# Clinical Significance of Carbapenem-Tolerant *Pseudomonas aeruginosa* Isolated in the Respiratory Tract

**DOI:** 10.3390/antibiotics9090626

**Published:** 2020-09-21

**Authors:** Momoyo Azuma, Keiji Murakami, Rina Murata, Keiko Kataoka, Hideki Fujii, Yoichiro Miyake, Yasuhiko Nishioka

**Affiliations:** 1 Devision of Infection Control and Prevention, Tokushima University Hospital, Tokushima 770-8503, Japan; azumm@tokushima-u.ac.jp; 2 Department of Respiratory Medicine and Rheumatology, Graduate School of Biomedical Sciences, Tokushima University, Tokushima 770-8501, Japan; yasuhiko@tokushima-u.ac.jp; 3 Department of Oral Microbiology, Institute of Biomedical Sciences, Tokushima University Graduate School, Tokushima 770-8501, Japan; p.aeruginosa.s2@gmail.com (R.M.); hfujii@tokushima-u.ac.jp (H.F.); miyake@tks.bunri-u.ac.jp (Y.M.); 4 Department of Microbiology and Genetic Analysis, Institute of Biomedical Sciences, Tokushima University Graduate School, Tokushima 770-8501, Japan; kataokakeiko@tokushima-u.ac.jp; 5 Department of Oral Health Sciences, Faculty of Health and Welfare, Tokushima Bunri University, Tokushima 770-8514, Japan

**Keywords:** carbapenem tolerance, antibiotic tolerance, *Pseudomonas aeruginosa*, respiratory tract infection, persistent infections

## Abstract

We often come across difficult to treat infections—even after administering appropriate antibiotics according to the minimal inhibitory concentration of the causative bacteria. Antibiotic tolerance has recently started to garner attention as a crucial mechanism of refractory infections. However, few studies have reported the correlation between clinical outcomes and antibiotic tolerance. This study aims to clarify the effect of antibiotic tolerance on clinical outcomes of respiratory tract infection caused by *Pseudomonas aeuginosa* (*P. aeruginosa*). We examined a total of 63 strains isolated from sputum samples of different patients and conducted a retrospective survey with the medical records of 37 patients with imipenem-sensitive *P. aeruginosa* infections. Among them, we selected 15 patients with respiratory infections, and they were divided into high-tolerance minimal bactericidal concentration for adherent bacteria (MBC^AD^)/minimal inhibitory concentration for adherent bacteria (MIC^AD^) ≥ 32 (*n* = 9) group and low-tolerance MBC^AD^/MIC^AD^ ≤ 16 (*n* = 6) group for further investigations. The findings indicated that the high-tolerance group consisted of many cases requiring hospitalization. Chest computed tomography findings showed that the disease was more extensive in the high-tolerance group compared to the low-tolerance group. Regarding the bacterial phenotypic characterization, the high-tolerance group significantly upregulated the production of the virulence factors compared to the low-tolerance group. Our study provided evidence that carbapenem tolerance level is a potent prognostic marker of *P. aeruginosa* infections, and carbapenem tolerance could be a potential target for new antimicrobial agents to inhibit the progression of persistent *P. aeruginosa* infections.

## 1. Introduction

*Pseudomonas aeruginosa* has become an important cause of infection, especially in immunocompromised patients [1,2]. *Pseudomonas* infections are complicated and can be severe and life-threatening. *P. aeruginosa* is also a cause of chronic respiratory tract infections [3]. Antibiotic tolerance is defined as the ability of a bacteria to survive, but not grow in the presence of antibiotic concentrations above the minimum inhibitory concentration (MIC) [4]. The minimum bactericidal concentration (MBC) denotes the minimum concentration required to kill 99.9% of the bacteria, whereas MIC is the lowest concentration of antibacterial agent that inhibits the growth. Treatment can be unsuccessful despite the selection of antibacterial agents on the basis of MIC, which could be because of antibiotic tolerance. In cases of antibiotic tolerance, wherein MIC and MBC get apart, complete elimination of the bacteria is not achieved despite suppressing bacterial growth.

Previous studies have reported that clinical isolates of Gram-positive or Gram-negative bacteria show tolerance to various kinds of antibiotics. This is one of the proposed explanations of poor response to antimicrobial therapies [5,6,7]. In addition, it is well known that *P. aeruginosa* normally attaches to a solid surface from the planktonic state to form a micro-colony and produces exopolysaccharides forming a biofilm. In usual non-mucoid type biofilms, *P aeruginosa* uses two types of exopolysaccharide (Psl and Pel), and Psl has a critical function in antibiotic tolerance in *P. aeruginosa* [8,9]. Few studies have evaluated the correlation between antibiotic tolerance and clinical outcomes. The aim of this study was to assess the effects of carbapenem tolerance on the clinical outcomes of respiratory tract infections caused by *P. aeruginosa* and investigate the participation of Psl in *P. aeruginosa* chronic infections.

## 2. Patients and Methods

### 2.1. Bacterial Strains

We used *P. aeruginosa* type strain PAO1 and retrospectively examined a total of 63 bacterial strains isolated from the sputum samples of different patients in Tokushima University Hospital from September 2011 to November 2013. Strain identification was carried out by an automatic biochemical identification system (MicroScan WalkAway, Beckman Coulter, Inc., Brea, CA, USA).

### 2.2. Susceptibility Testing for Planktonic Bacteria

The MIC and MBC of planktonic bacteria for Imipenem (IPM) (Wako Pure Chemical Industries, Ltd., Osaka, Japan) were assessed using a standard microbial broth dilution method [10].

### 2.3. Patient Recruitment

**Figure 1:**
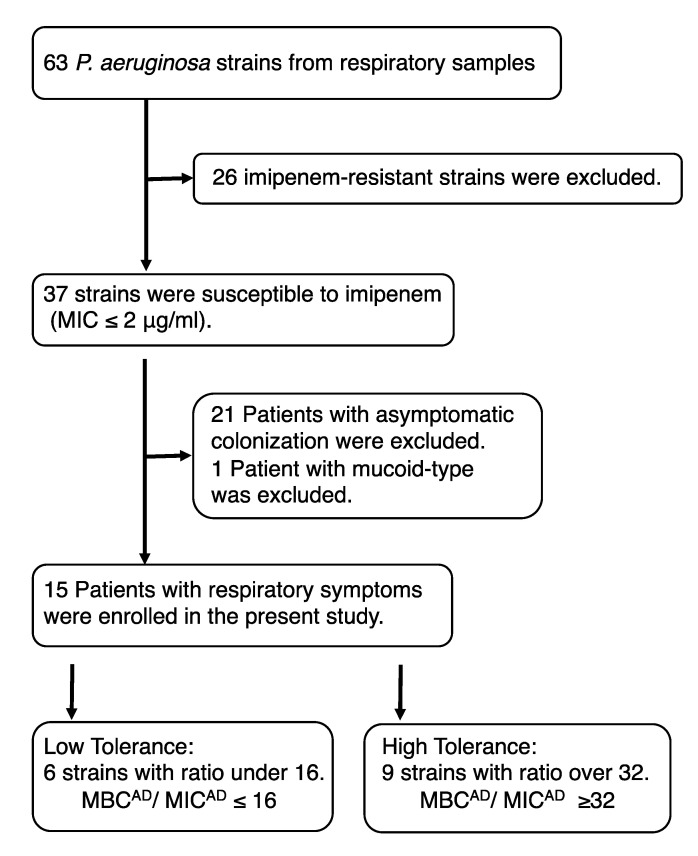
Flowchart of the patients included in this study. The low-tolerance and high-tolerance groups were defined (MIC, minimum inhibitory concentration; MBC, minimum bactericidal concentration; MIC^AD^, minimum inhibitory concentration of adherent bacteria; MBC^AD^, minimum bactericidal concentration of adherent bacteria).

We used the Clinical and Laboratory Standards Institute Guideline M100-S22 breakpoint to determine the sensitivity of *P. aeruginosa*. After obtaining sputum samples from the patients, we conducted a retrospective survey with the medical records of 37 patients with IPM sensitive *P. aeruginosa* infections. After excluding a patient with mucoid-type *P. aeruginosa* infection, the remaining 15 patients with chronic or severe respiratory symptoms were divided into low-tolerance (minimal bactericidal concentration for adherent bacteria (MBC^AD^)/minimal inhibitory concentration for adherent bacteria (MIC^AD^) ≤ 16 (*n* = 6) group and high-tolerance (MBC^AD^/MIC^AD^ ≥ 32 (*n* = 9) group for further investigations (Figure 1). In chest computed tomography (CT) findings, we defined the diffused type as abnormal findings spreading over more than two lobes of the lungs, and local type as abnormal findings localized in one lobe of the lung.

### 2.4. Susceptibility Testing for the Adherent Bacteria

The stationary phase liquid culture was diluted with saline to obtain a concentration of 2 × 10^6^ CFU/mL. A multi-well tissue culture plate (96-well, Falcon 3047, Becton Dickinson, Lincoln Park, NJ, USA; each well contained 50 μL of bacterial suspension) was centrifuged at 450× *g* for 15 min at 25 °C. After incubation at 37 °C for 1 h, saline was removed, and 100 μL of serially diluted antibiotic solutions were transferred to the wells from round-bottomed plates. The bacterial growth was assessed by visual inspection after 24-h incubation at 37 °C. MIC^AD^ was defined as the lowest concentration of antibiotic at which there was no bacterial growth. The antibiotic solutions were removed, and 200 μL of fresh LB medium (Thermo Fisher Scientific, Waltham, MA, USA) without antibiotics was added to each well, followed by further incubation for 24 h at 37 °C. MBC^AD^ was defined as the lowest concentration of antibiotic at which there was no bacterial growth [9].

### 2.5. In Vitro Phenotypic Characterization

Elastase production was assessed by Elastin Congo Red (ECR) (Elastin Products Company, Inc., Owensville, MO, USA) assay following the method of Pearson et al. [11]. Rhamnolipids production was evaluated using the following method: M9-based agar plates were used, and the diameter of the halo surrounding the colony was measured for rhamnolipids production [12]. For pyocyanin and pyoverdine production assay, the bacterial strains were grown in King A (peptone 20.0 g, glycerol 10.0 mL, K_2_SO_4_ 10.0 g, and MgCl_2·_6H_2_O 3.5 g) or B (peptone 20.0 g, glycerol 10.0 mL, K_2_HPO_4_ 1.5 g and MgPO_4·_7H_2_O 1.5 g) medium. The amounts of pyocyanin or pyoverdine in the culture supernatant were recorded by a spectrophotometer at 695 nm or by fluorescence emission at 460 nm after excitation at 400 nm [13].

The biofilms were grown by using the MBEC physiology and genetics assay kit (Innovotech Inc., Edmonton, AB, Canada) for 24 h at 37 °C with aeration [14]. The biofilms were washed and disrupted by sonication (Branson 3510, Branson Ultrasonics Corp., Danbury, CT, USA) for 1 h. The bacterial viability was measured by BacTiter-Glo Microbial Cell Viability Assay (Promega, Madison, WI, USA), and relative biofilm formation was calculated according to the value of PAO1. Swimming, swarming, and twitching assays were performed with 1% agar tryptone plates, 0.5% agar M8 plates supplemented with 0.2% glucose and 2mM MgSO_4_, and 1% agar LB plates as described previously [13,15].

### 2.6. Detection of the pslA Gene

For detection of the *pslA* gene, the primers pslA-s (CAGGCACTGGACGTCTACTC) and pslA-a (GTTGCGTACCAGGTATTCGG) were designed to amplify a 309-bp fragment within the coding sequence of the *pslA* gene [9]. PCR was performed in 20 μL of reaction mixture containing 50 ng of chromosomal DNA, 10 μL PrimeSTAR MAX Premix (Takara Bio Inc., Kusatsu, Japan), 10 pmol of each primers. PCR conditions for the amplification step were as follows: Initial denaturation at 98 °C for 30 s followed by 30 cycles of 98 °C for 10 s, 58 °C for 5 s and 72 °C for 5 s. DNA fragments were detected on 1.7% agarose gel stained with GelRed™ (Biotium Inc., Fremont, CA, USA).

### 2.7. Gene Expression of the pelA and pslA Genes

Bacteria were incubated at 37 °C with aeration in LB broth until the culture reached a log-phase. For analysis of gene expression in adherent cells, the log-phase culture was diluted with saline. Each well of a 6-well tissue culture plate (Becton Dickinson, Franklin Lakes, NJ, USA) received 1.5 mL of bacterial suspension, and the plate was centrifuged at 450× *g* for 15 min at 25 °C. After centrifugation, the saline was removed, and 500 μL of RNAprotect Bacteria Reagent (QIAGEN, Valencia, CA, USA) was added to the wells. The cells were removed with a cell scraper. For gene expression analysis of planktonic cells, the log-phase cells were used as a control. Total RNA was isolated using RNeasy kit (Qiagen). Reverse transcription was performed by using the SuperScript III First-Strand cDNA Synthesis System (Thermo Fisher Scientific, Waltham, MA, USA). qRT-PCR was performed using StepOnePlus™ Real-Time PCR System with Fast SYBR^®^ Green Master Mix (Thermo Fisher Scientific, Waltham, MA, USA). Expression was normalized to *rpsL*. The results were expressed as fold-change values relative to the control of planktonic cell samples [9].

### 2.8. Statistical Analyses

The data are presented as the mean ± standard deviation (SD). All comparisons between the populations were performed by Student’s *t*-test or Fisher’s exact test. All statistical analyses were performed by GraphPad PRISM 5.01 (GraphPad Software, Inc., La Jolla, CA, USA), and a *p* value of <0.05 was considered as statistically significant.

## 3. Results

### 3.1. MIC Distribution and Resistance Ratio

**Figure 2:**
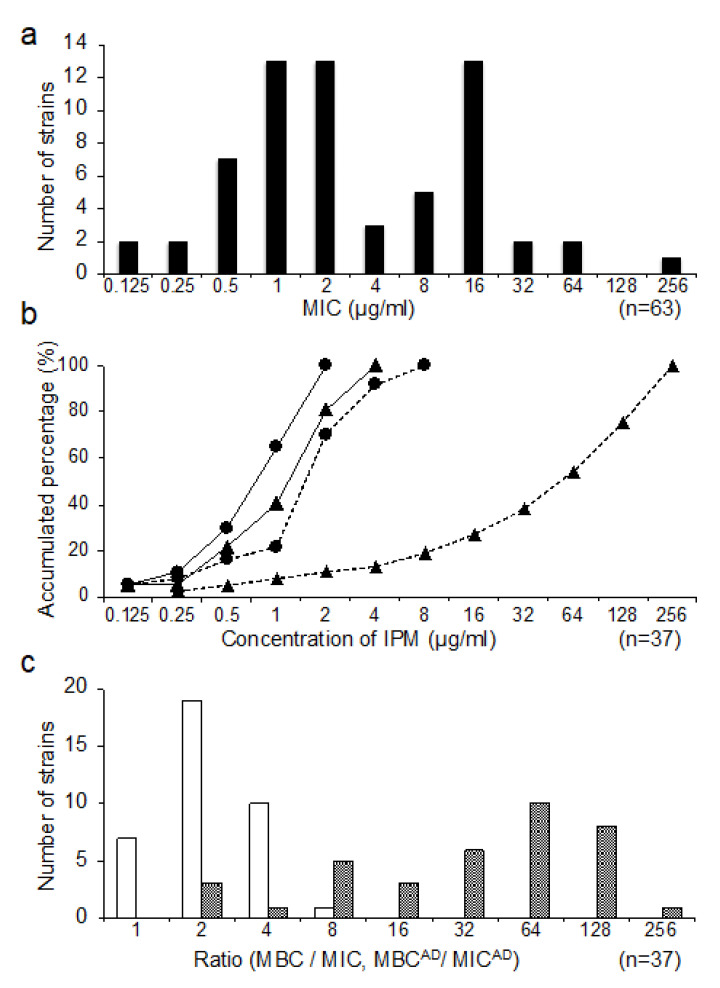
The MIC distribution for Imipenem of *Pseudomonas aeruginosa* clinical isolates from the respiratory apparatus samples (*n* = 63) (**a**). Accumulated percentage of MIC, MBC, MIC^AD^, and MBC^AD^ for IPM in the sensitive isolates (*n* = 37) (**b**). MIC (circles on the solid line), MBC (circles on the dotted line), MIC^AD^ (triangles on the solid line), and MBC^AD^ (triangles on the dotted line). The ratio distribution of planktonic bacteria (MBC/MIC) and adherent bacteria (MBC^AD^/MIC^AD^) in IPM-sensitive isolates (*n* = 37) are shown (**c**). The white bars represent the number of strains of MBC/MIC in planktonic bacteria, and the black bars represent the number of strains of MBC^AD^/MIC^AD^ in the adherent bacteria.

The MIC distribution for IPM is shown in Figure 2a. Out of the 63 clinical strains, 37 (58.7%) were sensitive (MIC ≤ 2 μg/mL). In this study, we focused on 37 sensitive strains to investigate antibiotic tolerance.

### 3.2. Distribution of MIC^AD^ and MBC^AD^

We estimated MIC^AD^ and MBC^AD^ of IPM for 37 IPM sensitive strains. Although the MIC^AD^ and MIC values were almost the same, the MBC^AD^ values were far higher than the MBC values for almost all the strains (Figure 2b). The ratio of MBC/MIC was obtained from 1 to 8 in planktonic cells; however, the ratio of MBC^AD^/MIC^AD^ was obtained from 2 to 256 in the adherent cells (Figure 2c).

### 3.3. Clinical Features

The clinical features of low- and high-tolerance groups are compared in Table 1. It was confirmed that the same clone did not exist among 15 clinical isolates in low- and high-tolerance groups by a phage-open reading frame typing (POT) (Cica Geneus^®^ Pseudo POT KIT, Kanto chemical Co., Tokyo, Japan) and Enterobacterial Repetitive Intergenic Consensus (ERIC)-PCR [16,17]. No significant difference was observed in age, gender, respiratory symptoms, comorbid-lung diseases, inflammatory markers, and baseline respiratory parameters. In the low-tolerance group, however, a significantly higher number of patients had underlying diseases, such as chronic sinusitis that could affect airway lesion-related symptoms (*p <* 0.05). In the low-tolerance group, although no significant difference was noted, the number of patients with underlying diseases, such as collagen diseases that could affect airway lesions, was higher. Furthermore, only in the high-tolerance group, two patients with diffuse panbronchiolitis (DPB) and two patients with chronic obstructive pulmonary disease (COPD) were identified. In chest CT, almost all the patients in the high-tolerance group had diffused abnormal findings spreading over more than two lobes of the lungs; however, in the low-tolerance group, they were mostly of local types (*p* < 0.05). The number of patients who required hospitalization, due to acute infection, was also significantly higher than in the high-tolerance group (*p* < 0.01), and one patient developed fatal *P. aeruginosa* sepsis. The value of SpO_2_, a parameter for respiratory failure, was lower in the high-tolerance group, and many patients were in a state of respiratory failure. The details of antibiotic administration in both groups are shown in Table 2. The use of macrolides for anti-inflammatory effects was excluded from the table. The amount of anti-pseudomonal antibiotics used was clearly high in the high-tolerant group. Few patients in either group had a history of treatment with carbapenem.

We showed each typical clinical case from the low- and high-tolerance groups:

#### Case 1: Low-tolerance

**Figure 3:**
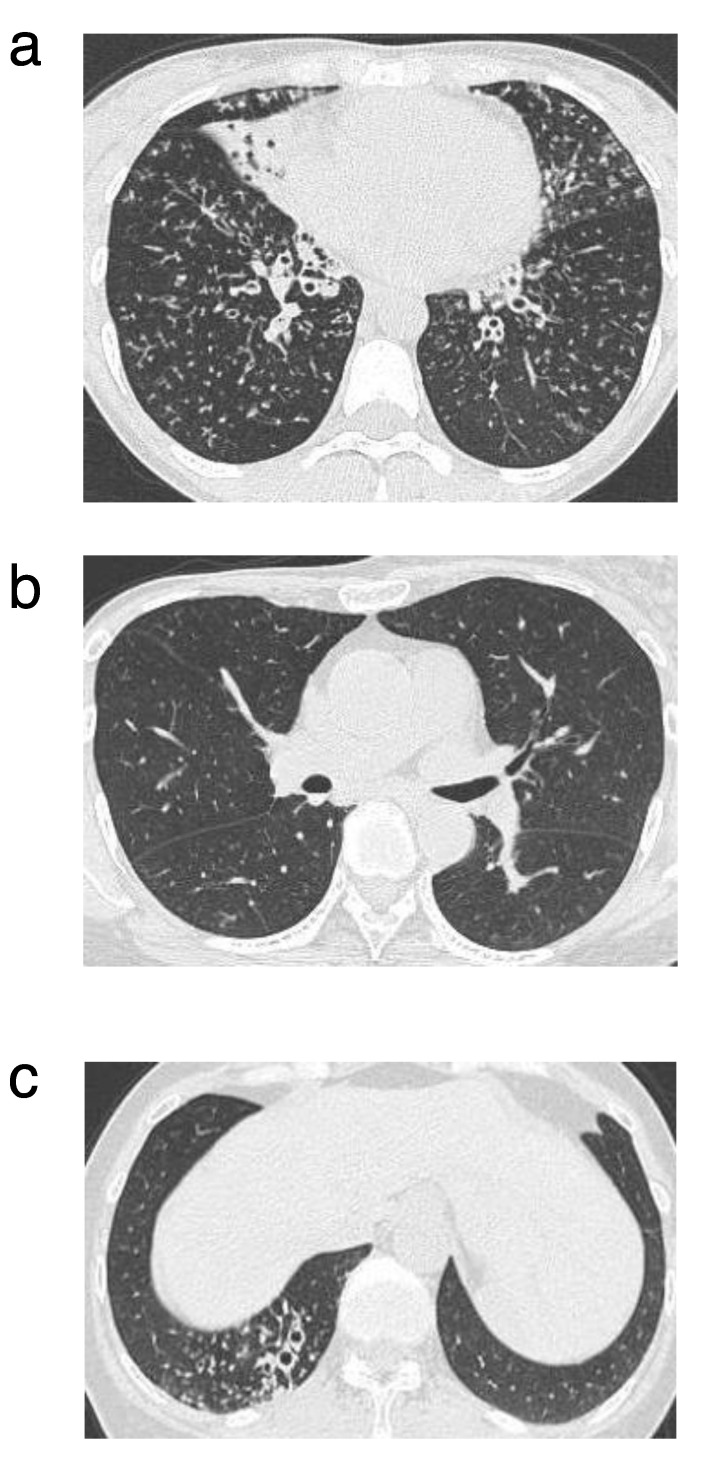
Chest computed tomography (CT) findings of the typical clinical cases from the low- and high-tolerance groups. The chest CT from the low- tolerance group showed only right lower lobe bronchiectasis and small nodules in 2012. The other lobes were normal (**a**,**b**). The chest CT from the high-tolerance group showed diffused small rounded and linear opacities, dilation of small bronchi, and bronchial wall thickenings in 2009. It was predominantly located in the lower lobe (**c**).

A 62-year-old woman visited our hospital in 2012 with a diagnosis of *bronchiectasis* and chronic bronchial infection, due to *P. aeruginosa*. Long-term clarithromycin therapy (200 mg/day) was initiated for its anti-inflammatory effects. The patient had a history of community-acquired pneumonia (CAP) and was treated with levofloxacin on an outpatient basis in 2012. Levofloxacin was used more than once every year for the treatment of acute exacerbation of bronchitis, and the symptoms were resolved effectively. Carbapenems were never administered to her. She did not have any respiratory failure, and her SpO_2_ was not low (98%) without any oxygen support. Pulmonary function test revealed normal results [VC, 3.16 L (%VC, 126); and FVC, 3.04 (L) (% FVC, 121.6)]. Chest CT showed only right lower lobe bronchiectasis and small nodules without significant degradation (Figure 3a,b). Bacterial phenotypic characterization showed antibiotic tolerance (MBC^AD^/MIC^AD^) was low (2) and relative biofilm formation to PAO1 was low (10%).

#### Case 2: High-tolerance

A 34-year-old woman visited our hospital in 2009 with diffuse panbronchiolitis (DPB) and chronic bronchial infection, due to *P. aeruginosa*. Long-term erythromycin therapy (400 mg/day) was initiated for its anti-inflammatory effects. Carbapenems were never administered to her. She had acute exacerbations over once a year. Specific respiratory symptoms (dyspnea, cough, and hemoptysis) were observed. The patient had respiratory failure, and her SpO_2_ was low (92%) without any oxygen support. Pulmonary function test performed in 2009 revealed the following results: VC, 1.5 (L) (%VC, 52.6); and FVC, 1.5 L (% FVC, 52.6). Chest CT showed diffused small rounded and linear opacities and dilation of small bronchi and bronchial wall thickenings (Figure 3c). In 2013, she was diagnosed with acute exacerbation, due to *P. aeruginosa* three times, and *P. aeruginosa* was isolated from sputum. At that time, there was no rapid deterioration, whereas the symptoms and imaging findings were gradually worsened. Bacterial phenotypic characterization showed antibiotic tolerance (MBC^AD^/MIC^AD^) was high (128), and relative biofilm formation to PAO1 was high (205%).

### 3.4. Bacterial Phenotypic Characterization

**Figure 4:**
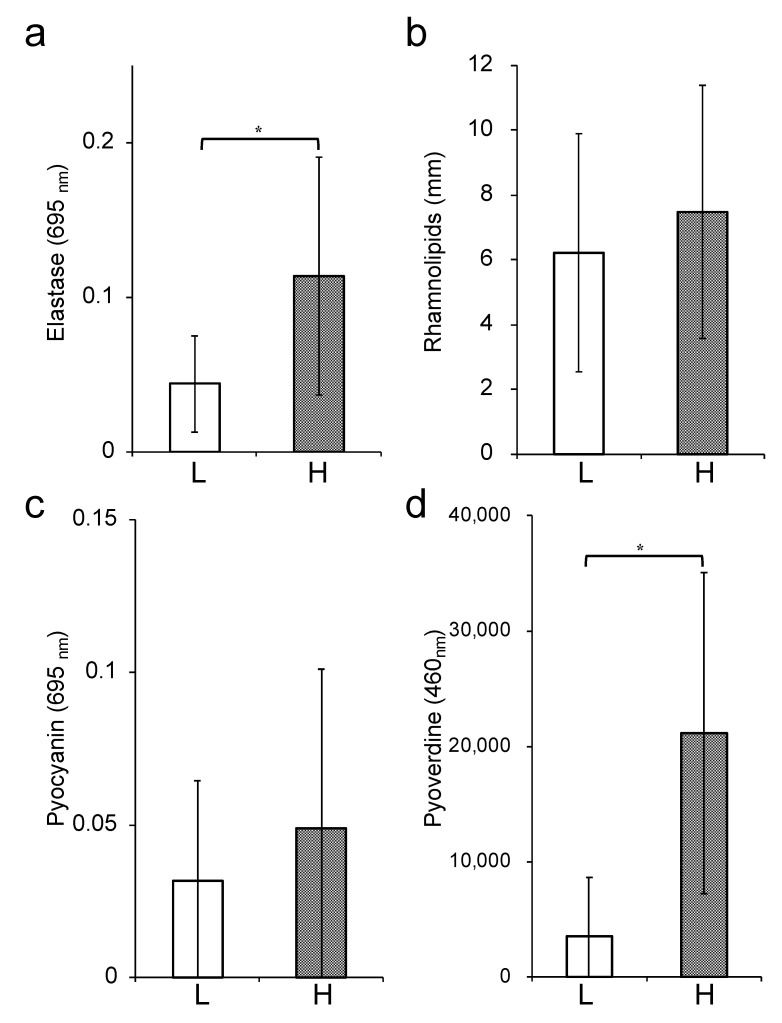
Bacterial virulent factors production in the low- and high-tolerance groups. Elastase production (**a**), rhamnolipid production (**b**), pyocyanin production (**c**), and pyoverdine production (**d**) are shown. The white bars represent the low-tolerance group (*n* = 6), and the black bars represent the high-tolerance group (*n* = 9). Each experiment was performed in triplicate. The data are presented as the mean ± SD for three experiments (* *p* < 0.05 compared to the low-tolerance group).

**Figure 5:**
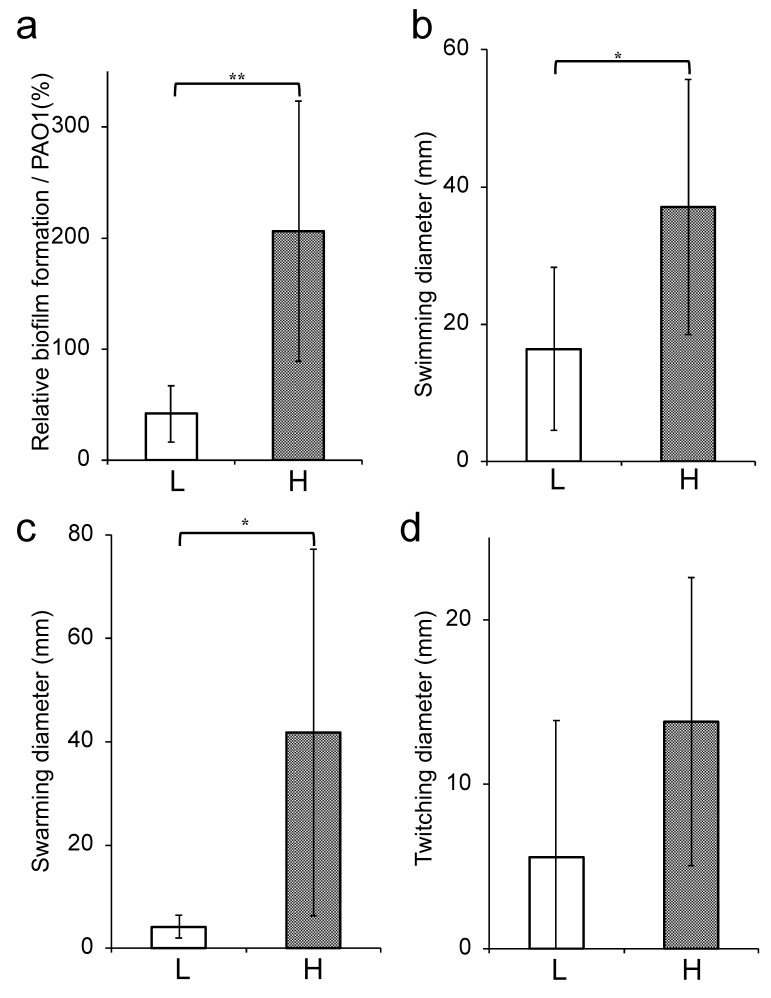
Bacterial biofilm formation and motility activities of the low- and high-tolerance groups. Relative biofilm formation (**a**), swimming motility (**b**), swarming motility (**c**), and twitching motility (**d**) are shown. The white bars represent the low-tolerance group (*n* = 6), and the black bars represent the high-tolerance group (*n* = 9). Each experiment was performed in triplicate. The data are presented as the mean ± SD for three experiments (* *p* < 0.05, ** *p* < 0.01 compared to the low-tolerance group).

Comparison of the bacterial phenotypic characterization between the low- and high-tolerance groups revealed that the production of elastase and pyoverdine as virulence factor was significantly upregulated in the high-tolerance group than the low-tolerance group (*p* < 0.05) (Figure 4a,d). On the other hand, no significant difference was observed between the two groups for pyocyanin and rhamnolipid production (Figure 4b,c). The ability to form biofilms was significantly higher in the high-tolerance group (206.1 ± 117.1) than the low-tolerance group (41.7 ± 25.3) (*p* < 0.01) (Figure 5a). Finally, we estimated swimming, swarming, and twitching activities that indicated the motility of *P. aeruginosa*. Although a significant difference was not noted in the twitching motility (Figure 5d), the swimming and swarming motilities were significantly higher in the high-tolerance group (37.0 ± 18.6 and 41.8 ± 35.5, respectively) than the low-tolerance group (16.4 ± 11.9 and 4.2 ± 2.2, respectively) (*p* < 0.05) (Figure 5b,c).

### 3.5. pslA Gene Detection and Transcription of pslA and pelA Genes in Adherent Cells

**Figure 6:**
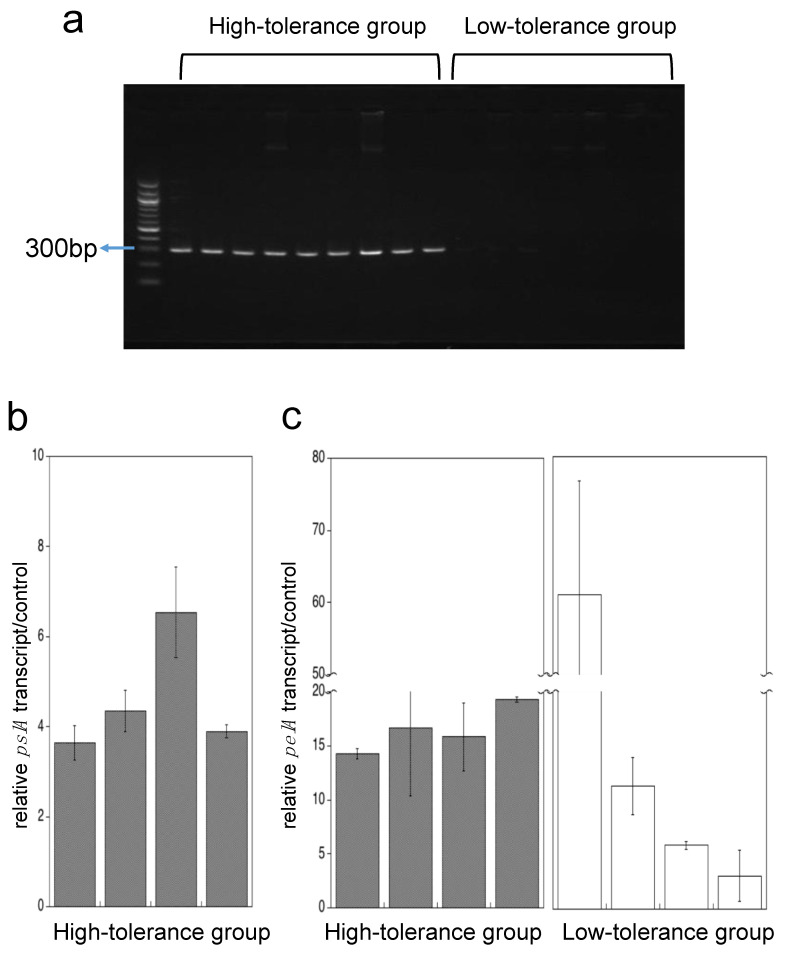
*pslA* gene detection and transcription of *pslA* and *pelA* genes in adherent cells. Agarose gel electrophoresis of *pslA* amplicon (309bp) (**a**). PCR products were detected using 1.7% agarose gel stained with GelRed™. *pslA* gene expression of adherent cells in the high-tolerance group (**b**), and *pelA* gene expression of adherent cells in the high- and low-tolerance group (**c**) are shown. The black bars represent the high-tolerance group (*n* = 4), and the white bars represent the low-tolerance group (*n* = 4). Each experiment was performed in triplicate. The data are presented as the mean ± SD for three experiments.

*pslA* gene was detected among all nine strains in the high-tolerance group, whereas in a low tolerance group was not detected at all (Figure 6a). The *pelA* gene was detected among all 15 strains in both groups (data not shown).

We used qRT-PCR to measure *pslA* or *pelA* transcript levels of adherent cells by using each four strains randomly selected from high- and low-tolerance group. In *pslA* expression, adherent cells had more than 4 times higher than that of planktonic cells in the high-tolerance group (Figure 6b). The *pelA* transcript level of adherent cells had increased more than four times compared to planktonic cells in all eight strains (Figure 6c).

## 4. Discussion

In the present study, we investigated the correlation between carbapenem tolerance and clinical features using isolates of *P. aeruginosa* obtained from sputum samples of patients. The findings indicated that the high-tolerance group included many severe cases with respiratory failure. Imaging results showed that the disease was more extensive in the high-tolerance group. Regarding the bacterial characteristics, the high-tolerance group bacteria showed significantly upregulated biofilm formation, elastase production, pyoverdine production, swimming motility, and swarming motility compared to the low-tolerance group.

In the clinical setting, MIC is one of the most important factors in the selection of antibiotics. We observed cases that were difficult to treat even though appropriate antibiotics, according to the MIC, were administered. The present study revealed that MBC^AD^ of IPM for adherent bacteria was far higher than MIC^AD^, demonstrating that *P. aeruginosa* has an extremely high potential tolerance to antibiotics. This tendency was almost the same with meropenem (data not shown). Caution must be exercised with bacterial strains that exhibit extremely high antibiotic tolerance because recurrence can occur during the course of treatment.

One of the most alarming problems caused by *P. aeruginosa* is related to its ability to cause chronic infection in patients with respiratory disease, such as DPB or COPD. Colonization of *P. aeruginosa* in these patients is associated with increased mortality [18,19,20]. DPB is characterized by chronic inflammation resulting from a main lesion within the respiratory bronchioles. It is diffused in both the lungs and lead to respiratory disorders. COPD is a progressive, debilitating lung disorder characterized by non-normalizing airflow limitation [21]. Notably, two DPB cases and two COPD cases were reported in the high-tolerance group, but none was reported in the low-tolerance group in this study. The adherence of high-tolerant *P. aeruginosa* to the respiratory tract with decreased ciliary function results in a chronic infection leading to reduced respiratory function over time.

In the low-tolerance group, although the strains exhibited low virulence, chronic infections did occur. This may be due to host factors, such as sinusitis or autoimmune disease. However, the clinical manifestations did not get worse and recovered only by outpatient-based treatment, possibly due to low antibiotic tolerance and virulence.

In the bacterial phenotypic characterization assay, biofilm formation was significantly increased in the high-tolerance group compared to the low-tolerance group. In the low-tolerance group, low biofilm formation certainly resulted from the deficiency of the *pslA* gene. It is well known that biofilms evade antimicrobial challenges by several mechanisms, such as failure of penetration, reduced susceptibility, etc. [22,23]. Swimming is dependent on flagellar motility in liquid media, while twitching depends on type VI fimbria motility with surface adherence and swarming comprises both forms of motility [24]. In the present study, the results of the motility assay suggested that the bacteria in the high-tolerance group had strong flagellar motility. The attachment to the surface of *Vibrio alginolyticus* is swimming speed-dependent [25]. The mobility required to attach to the surface could be strongly related to biofilm formation. From this point, therefore, it is rational to think that the bacteria in the high-tolerance group exhibited higher biofilm formation and flagellar activity. Some signals transmitted under appropriate conditions triggered dispersal of biofilm cells back to the planktonic state causing acute infection [26], and flagellar activity could be related to the dispersion frequency of the biofilm cells.

The bacteria in the high-tolerance group produced increased amounts of pyoverdine and elastase than that in the low-tolerance group. Pyoverdine is one of the major siderophores presented in *P. aeruginosa* and plays a crucial role in acquiring iron, which is necessary for bacterial growth, biofilm formation, and infection [27,28,29]. Elastase is an important virulence factor that damages host tissues [30,31]. The data of the present study suggested that antibiotic tolerance was positively correlated to the virulence and was possibly regulated by the same mechanisms.

The quorum-sensing system is known to be an important regulator of virulence: Biofilm formation, pyocyanin, pyoverdine, elastase, and rhamnolipid production [32]. Quorum-sensing-dependent virulence traits are important for the development of infection. Quorum-sensing molecules were detected in sputum or bronchoalveolar lavage fluid [33,34]. In the present study, antibiotic tolerance was not related to pyocyanin and rhamnolipid productions. Antibiotic tolerance in QS knockout mutant (PAO1Δ*lasI*, *rhlI*) was not different from that of the PAO1 (data not shown). These results suggested that antibiotic tolerance was not directly regulated by quorum-sensing.

In our results, *pelA* expression was induced by surface adherence in all eight strains. The transcription of *pel* is directly regulated by c-di-GMP through FleQ, which is a transcriptional regulator [35,36]. Thereby, surface adherence could induce intracellular c-di-GMP levels both in high- and low-tolerance groups. We recently suggested that the *psl* genes, which are activated by surface adherence through elevated intracellular c-di-GMP levels, confer tolerance to antimicrobials [9]. Psl might serve an integral role in *P. aeruginosa* severe chronic infections because the *psl* gene was upregulated by surface adherence in the high-tolerant group and was not detected in the low-tolerant group. Our work demonstrates that Psl and c-di-GMP signaling pathways induced by surface adherence in *P. aeruginosa* are prospective targets for new antibacterial agents.

In this study, *P. aeruginosa* strains in the high-tolerance group could exhibit higher virulence than that of the low-tolerance group. As few patients were treated with carbapenems in the high-tolerance group, it was not probable that the high carbapenem-tolerant strains were selected in the lungs after long-term administration of antibiotics. We speculated that the high-tolerance strains existing in the environment were transmitted to the patients and resulted in higher virulence and further exacerbated the clinical outcome. Our results indicated that carbapenem tolerance of the adherent cells might be a clinically useful predictive marker of *P. aeruginosa* infections.

In the same period, data from pneumonia patients isolated with IPM-resistant *P. aeruginosa* were retrospectively analyzed, and 19 patients were assessed. Eleven patients were treated (57.9%) with mechanical ventilation, and eight patients (42.1%) were dead. This result suggested that pneumoniae caused by carbapenem-resistant *P. aeruginosa* were much severer than that by the high-tolerance group. However, pneumonia in the high-tolerance group could be chronic, and we need to pay attention to the selection of antibiotics and the follow-up of the clinical course with pneumonia caused by *P. aeruginosa* in the high-tolerance group.

There are some limitations to the present study. First, this was a retrospective study that certainly involved a selection bias because of the nature of the study design. Second, a small cohort of patients from a single institution was included, which limited the generalizability of the study results. Further prospective studies in a higher number of patients are required to validate our findings and further investigate the respective mechanisms.

## 5. Conclusions

In conclusion, the carbapenem tolerance level is a useful predictive marker in *P. aeruginosa* infections, and furthermore, Psl, and c-di-GMP signaling pathway could be a new drug target for persistent *P. aeruginosa* infection.

## 6. Ethics Approval and Consent to Participate

The study was performed in accordance with the Declaration of Helsinki, and the study protocol was approved by the Institutional Review Board of Tokushima University Hospital (approval no. 1300-1). According to the Japanese Governmental Ethical Guidelines, we provided information on the purpose of the utilization of human biological specimens and provided opportunities to the possible research subjects to deny participating in the research instead of obtaining written informed consent. These procedures were approved by the Ethical Committee of Tokushima University Hospital.

## Figures and Tables

**Table 1:** Clinical features of low- and high-tolerance group.

Characteristics	Low-Tolerance (*n* = 6)	High-Tolerance (*n* = 9)	*p* Value
Age (years)	55 ± 27	55 ± 26	
Male sex	2 (33)	7 (78)	0.136
Pseudomonas infection			
Acute pneumonia	1 (17)	3 (33)	0.604
Persistent infection	5 (83)	6 (67)	0.604
Respiratory symptoms			
Cough/Sputum	6 (100)	9 (100)	1.00
Comorbidities			
Solid tumor	0 (0)	2 (22)	0.486
Autoimmune disease	3 (50)	1 (11)	0.235
Sinusitis	3 (50)	0 (0)	0.044 *
Congenital heart disease	1 (17)	1 (11)	1.00
NTM	1 (17)	0 (0)	0.341
Underlying lung diseases			
Bronchiectasis	5 (83)	4 (44)	0.286
DPB	0 (0)	2 (22)	0.486
COPD	0 (0)	2 (22)	0.486
Chest CT finding			
Diffuse type	1 (17)	7 (78)	0.041 *
Local type	5 (83)	2 (22)	0.041 *
Inflammatory markers			
CRP (mg/mL)	1.5 ± 1.5	4.0 ± 6.2	0.319
WBC (10^3^/μL)	6.6 ± 2.4	8.8 ± 5.0	0.362
Patients requiring hospitalization	1 (17)	8 (89)	0.011 *
No. of dead patients	0 (0)	1 (11)	1.00
Baseline respiratory parameters			
SpO_2_ (%)	97 ± 1	95 ± 3	0.21
Oxygen inhalation	0 (0)	3 (33)	0.229
Respiratory failure	0 (0)	4 (44)	0.103

NTM, non-tuberculous mycobacterium; DPB, diffuse panbronchiolitis; COPD, chronic obstructive pulmonary disease; Data are presented as means ± SDs or number of patients. Numbers in parentheses represent percentages among all patients. * *p* < 0.05 in comparison to Low-tolerance group.

**Table 2:** History of antibiotics.

Characteristics	Number of Patients in Low-Tolerance (Total Days)	Number of Patients in High-Tolerance (Total Days)
β-lactams		
Ampicillin	0	1 (7)
Sulbactam/Ampicillin	0	2 (40)
Clavulanic acid/Amoxicillin	0	2 (6)
Tazobactam/Piperacillin	0	1 (4)
Cefcapene pivoxil	3 (26)	2 (7)
Cefazolin	0	1 (6)
Cefmetazole	0	2 (13)
Ceftriaxone	1 (10)	0
Cefozopran	0	1 (5)
Sulbactam/Cefoperazone	1 (5)	0
Ceftazidime	0	3 (35)
Meropenem	1 (5)	1 (3)
Quinolones		
Levofloxacin	1 (21)	1 (4)
Garenoxacin	0	2 (12)
Moxifloxacin	0	1 (7)
Macrolides		
Clarithromycin	0	1 (5)
Clindamycin	0	1 (3)
Tetracycline		
Minocycline	0	1 (5)

Data are presented as total number of patients. Numbers in parentheses represent total days antibiotics had been administered.
